# Effect of Combination Therapy with Ezetimibe and Statins versus Statin Monotherapy on Carotid Intima-Media Thickness: A Systematic Review and Meta-Analysis of Randomized Controlled Trials

**DOI:** 10.3390/medicina59111980

**Published:** 2023-11-10

**Authors:** Ryuk Jun Kwon, Young Hye Cho, Eun Ju Park, Youngin Lee, Sae Rom Lee, Jung In Choi, Sang Yeoup Lee, Soo Min Son

**Affiliations:** 1 Family Medicine Clinic and Research Institute of Convergence of Biomedical Science and Technology, Pusan National University Yangsan Hospital, Yangsan 50612, Republic of Korea; brain6@hanmail.net (R.J.K.); younghye82@naver.com (Y.H.C.); everblue124@hanmail.net (E.J.P.); ylee23@gmail.com (Y.L.); sweetpea85@naver.com (S.R.L.); s1jungin@hanmail.net (J.I.C.); saylee@pnu.edu (S.Y.L.); 2 Department of Family Medicine, Pusan National University School of Medicine, Yangsan 50612, Republic of Korea

**Keywords:** ezetimibe, atherosclerosis, intima-media thickness, cardiovascular disease, stroke

## Abstract

*Background and Objectives*: Lipid-lowering agents such as ezetimibe are recommended in uncontrolled hyperlipidemia for primary and secondary prevention of cardiovascular disease. Carotid intima-media thickness (CIMT) is a surrogate marker of atherosclerosis and a predictor of cardiovascular and cerebral events. The effects of ezetimibe on CIMT have been inconsistently reported. The aim of this meta-analysis is to compare the effects of ezetimibe/statin and statin alone therapies on CIMT reduction. *Materials and Methods*: The PubMed, Embase, and Cochrane library databases were searched for randomized controlled trials (RCTs) published prior to 26 January 2023 with the MeSH keywords ‘Ezetimibe’ and ‘Carotid Intima-Media Thickness’. The results were presented as standard mean difference (SMD) with 95% confidence intervals using the random-effect model method, and heterogeneity was assessed. Subgroup, meta-regression, and sensitivity analyses were conducted. *Results*: Five RCTs with 642 participants were included. CIMT reduction was not significantly different between the ezetimibe/statin and statin alone groups. However, in subgroup analyses, CIMT in the ezetimibe/statin group was significantly reduced in patients with non-familial hypercholesterolemia (SMD: −0.34 mm and *p* = 0.002) and in patients with secondary prevention (SMD: −0.38 mm and *p* = 0.002). The low-density lipoprotein cholesterol level was significantly reduced in the ezetimibe/statin group (SMD: −0.58 mg/dL and *p* < 0.001). *Conclusions*: The effect of ezetimibe on CIMT reduction was shown in non-familial hypercholesterolemia and secondary prevention. These results suggest that the efficacy of ezetimibe may vary with potential CIMT reduction benefits in certain subpopulations.

## 1. Introduction

Statins are one of the recommended treatment options for dyslipidemia and are used for the primary and secondary prevention of cardiovascular disease (CVD) [1]. Several guidelines and studies recommend high-intensity statins for patients who are incapable of attaining the target lipid levels [2]. However, an increase in the dose of statins alone does not achieve the recommended target low-density lipoprotein cholesterol (LDL-C) levels [3], as only a 6% reduction in LDL-C can be achieved by doubling the statin dose [4]. Thus, the addition of other LDL-C lowering agents is useful for further LDL-C level reduction [5]. Ezetimibe, which inhibits intestinal cholesterol absorption by selectively binding to the Niemann–Pick C1-like 1 protein, reduces excessive LDL-C levels by an average of 23–24% when used in combination with statins [6]. It exhibited an anti-lipid effect that significantly lowered the occurrence of CVD events, as presented in studies of patients with aortic stenosis (Simvastatin and Ezetimibe in Aortic Stenosis trial), chronic kidney disease (Study of Heart and Renal Protection trial), and acute coronary syndrome (Improved Reduction of Outcomes: Vytorin Efficacy International Trial, IMPROVE-IT) [1].

Carotid intima-media thickness (CIMT)—the thickness of the intimal and medial layers of the walls of carotid arteries—can be measured simply and non-invasively using B-mode carotid ultrasound [7]. CIMT is known as a surrogate marker of atherosclerosis and as a powerful predictor of CVD [8]. According to a meta-analysis of 119 trials, a reduction in CIMT progression significantly reduces the CVD risk, and CIMT is a useful marker for assessing the extent of reduction in CVD risk through interventions such as lipid lowering as well as anti-hypertensive or anti-diabetic agents [9]. Moreover, CIMT showed a positive association with the incidence of stroke in a study of 5028 participants without stroke and CVD [10]. In particular, according to a meta-analysis of 26 trials that showed the effect of statins on stroke prevention, every 10% reduction in LDL caused by the use of statins decreases the risk of stroke by 15.6% and CIMT by 0.73%/year [11].

Several studies have investigated whether ezetimibe reduces the progression of CIMT [12,13,14,15,16]. However, consensual results were not obtained. Therefore, the objective of the present study is to determine whether the combined use of ezetimibe with a statin affects CIMT progression compared to the use of a statin alone through a meta-analysis of randomized controlled trials (RCTs).

## 2. Materials and Methods

We performed this systematic review according to the preferred reporting items for systematic reviews and meta-analyses (Table S1). Database search, study selection, assessment of quality, and data extraction were conducted by two authors (SMS and RJK) independently. This meta-analysis was not registered at PROSPERO.

### 2.1. Data Sources and Searching Strategy

A systematic review of the literature was performed using PubMed, Embase, and Cochrane Central Register of Controlled Trials from inception to 26 January 2023. The search was not restricted by language and the following MeSH keywords were used for the search: ‘Ezetimibe’ and ‘Carotid Intima-Media Thickness’ (Table S2).

### 2.2. Study Selection, Data Extraction, and Assessment of Quality

The criteria for studies to be included in this review were as follows: (1) RCTs; (2) comparison of the combined use of ezetimibe and a statin with the use of a statin alone; (3) results of CIMT at baseline and the end of the trial or CIMT change from baseline to end point; and (4) follow-up period of at least 12 months.

The titles and abstracts of all studies found in literature search were screened for suitability, and related articles were searched. After obtaining the full texts of all relevant studies, only RCTs were evaluated based on the outcome of interest. Initially, 27 trials were included in the meta-analysis, but 5 trials were analyzed because we only included RCTs with the same dosage of statins in both ezetimibe/statin and statin groups to determine the unique effect of ezetimibe on CIMT.

Two authors (SMS and RJK) independently extracted and summarized the data from eligible trials using standardized formats. The following information was extracted from each study: first author’s name with publication year, country, study duration, mean age of the participants, sample sizes of the intervention and control groups, regimen of lipid-lowering agents used (ezetimibe with a statin (ezetimibe/statin) and a statin only (statin alone)), and mean and standard deviation of CIMT, LDL-C, triglyceride (TG), and high-density lipoprotein cholesterol (HDL-C) at baseline. To ensure consistency in CIMT measurements, the average or maximum CIMT was extracted. The maximum CIMT was extracted when mean CIMT was not reported in the studies.

To assess the quality of all included studies, the Cochrane Collaboration’s tool was used for evaluating the risk of bias in the Review Manager Version 5.4.1 (Oxford, UK). Figure S1 shows the risk of bias including selection, performance, detection, reporting, and attribution.

### 2.3. Statistical Analysis

The combined results were expressed as standard mean difference (SMD) with 95% confidence interval (CIs) determined using the random-effect model method in Review Manager 5.4.1. Statistical heterogeneity was assessed using I-squared (I^2^) and Q statistics. Heterogeneity was statistically significant at *p* < 0.05. Subgroup analyses were performed according to presence of familial hypercholesterolemia (FH) and type of prevention to evaluate the potential source of heterogeneity among studies. In addition, lipid profile elements such as LDL-C, TG, and HDL-C levels were compared across FH- and non-FH-based subgroups to evaluate the effect of ezetimibe on CIMT. To identify the effect of each study on the combined effect size, sensitivity analysis was performed by removing one study at a time from the analysis, and to determine the reasons for the heterogeneity between studies, meta-regression was also conducted using the R software package version 4.2.1.

## 3. Results

### 3.1. Literature Search

**Figure 1:**
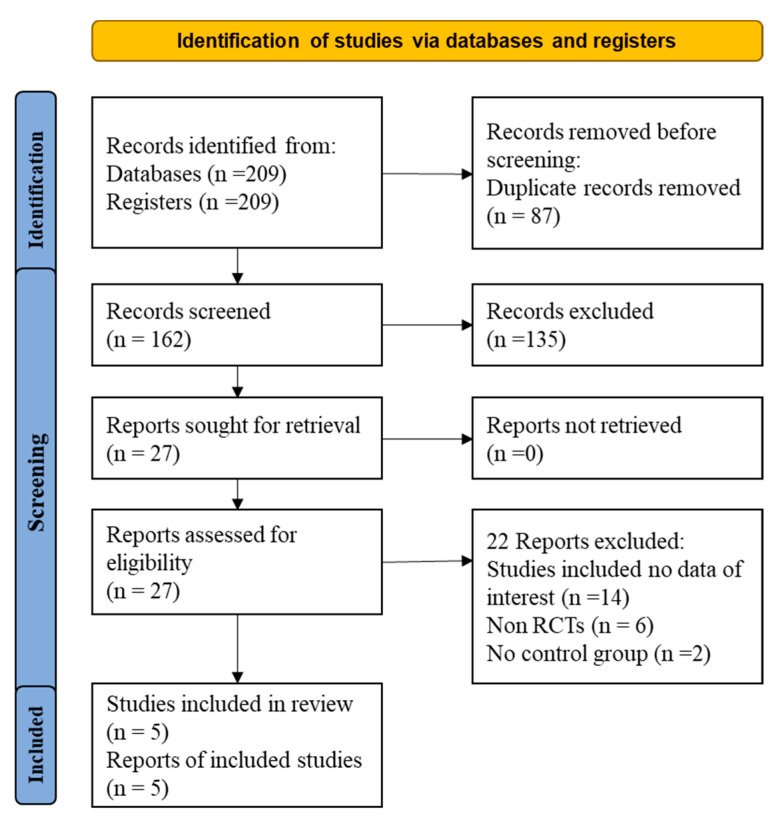
Flow diagram for the studies evaluated and included in systemic review and meta-analysis of randomized controlled trails.

Initially, 209 articles were found through the literature search (Figure 1). From them, 87 duplicate articles were removed. Of the 162 remaining papers, 135 were excluded after reading the title and abstract. After that, 22 of the remaining 27 articles were excluded for the following reasons (Table S3): (1) non-RCT study design or (2) insufficient data on the dosage of statins and/or ezetimibe. Finally, five articles were included in the present meta-analysis.

### 3.2. Characteristics of Included Studies

The trials were conducted in different countries (Netherlands, China, and Japan) as summarized in Table 1. Most of them had a study duration of 12 months, except the trial by Kastelein et al. [13]. The studies included patients with FH, coronary heart disease, type 2 diabetes, and non-familial hypercholesterolemia (non-FH). The sample size of the studies ranged from 54 to 720 patients. The dosage of ezetimibe was 10 mg/day in each study.

### 3.3. Changes in CIMT

**Figure 2:**
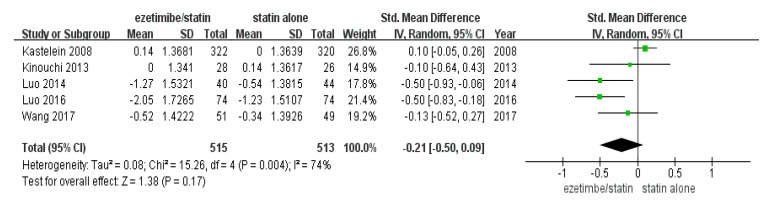
Forest plot: the effects of ezetimibe/statin on carotid intima-media thickness compared to statin alone. Std: standard; SD: standard deviation; CI: confidence interval; and I^2^: I-squared; green square: average for each study, black rhomb: overall average [13,15,17,18,19].

The total number of study participants enrolled in this meta-analysis was 1028 (intervention = 515 and control = 513) (Table 1). As shown in Figure 2, the random-effect model showed that CIMT was not significantly reduced in the ezetimibe/statin group compared to the statin alone group (*p* = 0.17) with substantial statistical heterogeneity (I^2^ = 74% and *p* = 0.004).

### 3.4. Subgroup Analyses to Estimate the Effects on CIMT in the Studies

**Figure 3:**
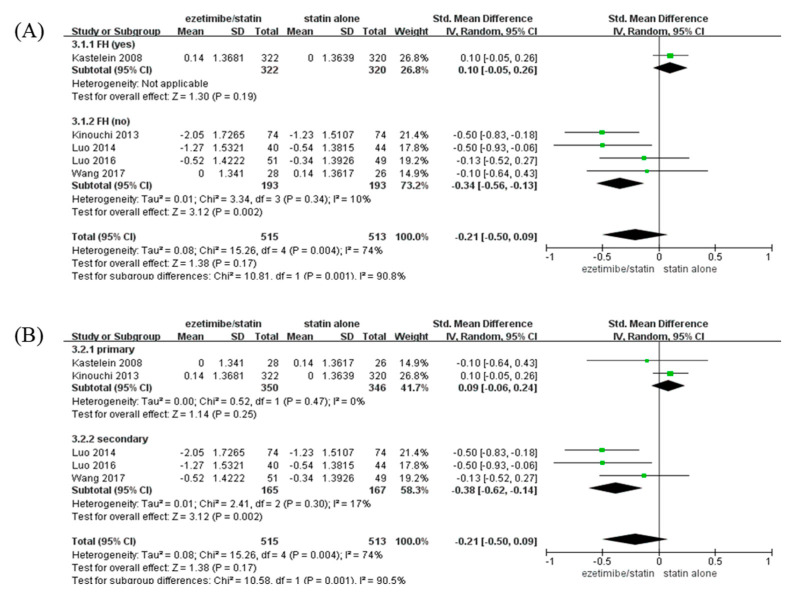
Subgroup analyses: the effects of ezetimibe on carotid intima-media thickness. (**A**) Subgroup analysis depending on the presence or absence of FH. (**B**) Subgroup analysis depending on prevention type—primary, or secondary prevention. Std: standard; SD: standard deviation; CI: confidence interval; I^2^: I-squared; and FH: familial hypercholesterolemia; green square: average for each study, black rhomb: overall average average [13,15,17,18,19].

CIMT of participants with non-FH in the ezetimibe/statin group was significantly reduced compared to that of participants in the statin alone group (SMD: −0.34 mm and *p* = 0.002) with low heterogeneity (I^2^ = 10% and *p* = 0.34). On the contrary, CIMT of participants with FH in the ezetimibe/statin group was not affected significantly (Figure 3A).

When analysis was performed according to prevention type, CIMT of participants with secondary prevention in the ezetimibe/statin group was significantly reduced compared to that of participants in the statin alone group (SMD: −0.38 mm and *p* = 0.002) with low heterogeneity (I^2^ = 17% and *p* = 0.30). In contrast, CIMT of participants with primary prevention in the ezetimibe/statin group was not affected statistically (SMD: 0.09 mm and *p* = 0.25) with no heterogeneity (I^2^ = 0% and *p* = 0.47) (Figure 3B).

### 3.5. Lipid Profile (LDL-C, TG, and HDL-C Levels) and Adverse Events

**Figure 4:**
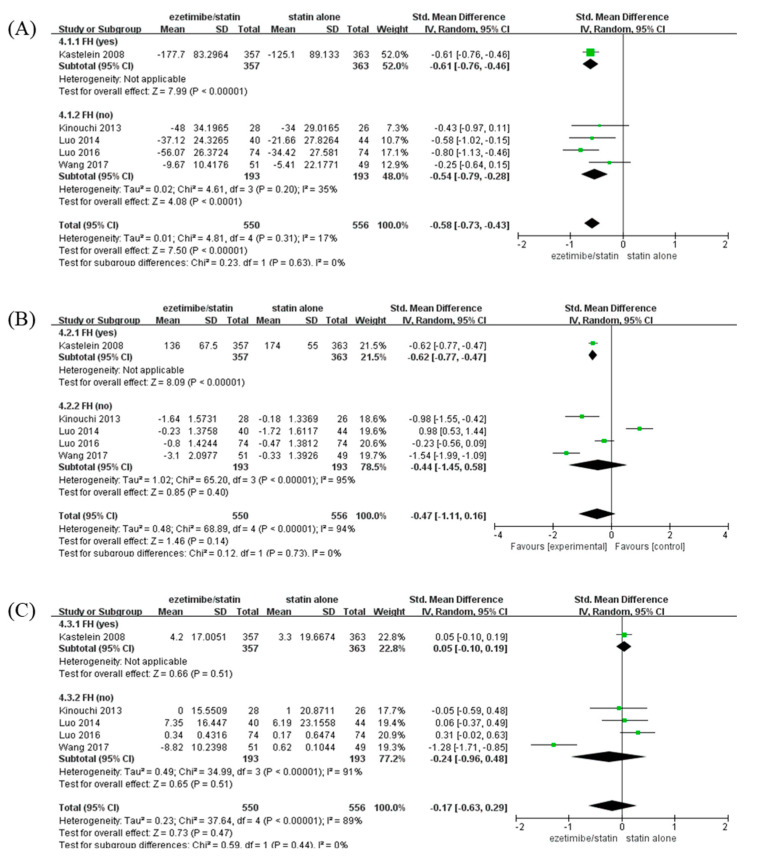
Forest plots: the effects of ezetimibe/statin on lipid profile compared to statin alone, established by presence or absence of FH. (**A**) LDL-C, (**B**) TG, and (**C**) HDL-C. Std: standard; SD: standard deviation; CI: confidence interval; I^2^: I-squared; LDL-C: low-density lipoprotein cholesterol; TG: triglyceride; HDL-C: high-density lipoprotein cholesterol; and FH: familial hypercholesterolemia; green square: average for each study, black rhomb: overall average [13,15,17,18,19].

To identify the changes in lipid profile caused by ezetimibe between the FH- and non-FH-based subgroups, changes in LDL-C, TG, and HDL-C levels were analyzed when ezetimibe/statin and statin alone were administered (Figure 4). The LDL-C level was significantly reduced in the ezetimibe/statin group compared to that in the statin alone group (SMD: −0.58 mg/dL and *p* < 0.001) with low heterogeneity (I^2^ = 17% and *p* = 0.31) (Figure 4A). In addition, both participants with FH and non-FH showed a significant reduction in LDL-C level in the ezetimibe/statin group (FH; SMD: −0.61 mg/dL and *p* < 0.001/non-FH; SMD: −0.54 mg/dL and *p* < 0.001). The TG level was not significantly different between the ezetimibe/statin and statin alone groups (SMD: −0.47 mg/dL and *p* = 0.14) with high heterogeneity (I^2^ = 94% and *p* < 0.001) (Figure 4B). However, the FH subgroup showed a significant reduction in TG level in the ezetimibe/statin group (SMD: −0.62 mg/dL and *p* < 0.001). Finally, the HDL-C level in the ezetimibe/statin group was not affected significantly (SMD: −0.17 mg/dL and *p* = 0.47) with substantial heterogeneity (I^2^ = 89% and *p* < 0.001) (Figure 4C). Several adverse events were assessed, although the same types of adverse events were not evaluated in all trials. When comparing cardiovascular events and side effects related to the liver, muscle, kidney, heart, and inflammation, which are commonly evaluated, there was no significant difference between the combination therapy and the statin monotherapy groups in the included studies (Table S4).

### 3.6. Sensitivity Analysis, Meta-Regresson, and Publication Bias

We performed sensitivity analysis to confirm the effect of each study on the combined effect size by removing one study at a time (Figure S2, Table S5). When the study by Kastelein et al. was removed from the analysis, the ezetimibe/statin group showed significantly reduced CIMT with lower heterogeneity (SMD: −0.34 mm, 95% CI: −0.56 to −0.13, *p* < 0.01, and I^2^: 10%). No significant effect of omitting other individual studies was evident on the combined effect size. In addition, meta-regression analysis was conducted with baseline CIMT (Figure S3A), baseline LDL (Figure S3B), baseline TG (Figure S3C), and baseline HDL (Figure S3D) as covariates to explain the heterogeneity of this study. The regression coefficients of these covariates (baseline CIMT and LDL) were significant, with values of −0.8063 and 0.0025 (*p* = 0.0034 and *p* < 0.001), respectively. R^2^ was 91.16% and 93.47%, respectively. However, we could not assess for publication bias based on Cochrane recommendations because fewer than ten trials were included.

## 4. Discussion

The combination therapy with ezetimibe and statins after acute coronary syndromes (ACS) is known to reduce the risk of death from cardiovascular causes such as a major coronary event and nonfatal stroke [6]. A recent study showed that the upfront combination treatment reduced all-cause mortality more than the guideline-based statin monotherapy in patients with ACS [20]. Moreover, in patients with DM, adding ezetimibe to a statin reduced acute ischemic events (myocardial infarction and ischemic stroke) compared to a statin alone. Therefore, ezetimibe is well established as an addition to statins for uncontrolled hyperlipidemia [21].

CIMT is known as a surrogate marker for atherosclerosis and a strong predictor of CVD and stroke [8]. Thus, ezetimibe, which is used to treat dyslipidemia when statin therapy is ineffective, has been investigated for its potential to decrease CIMT progression. The ENHANCE study (Ezetimibe and Simvastatin in Hypercholesterolemia Enhances Atherosclerosis Regression Trial), conducted in patients with heterozygous familial hypercholesterolemia, showed that the combination therapy of ezetimibe and simvastatin effectively lowered LDL cholesterol levels but had little impact on CIMT [13]. On the other hand, the SANDS (Stop Atherosclerosis in Native Diabetics Study), which aimed to prevent cardiovascular disease in diabetic patients, found that the combination therapy of ezetimibe and statin (aggressive group) was more effective in reducing CIMT compared to a statin alone (conventional group), although the change in CIMT between the statin/ezetimibe combination therapy and the statin monotherapy within the aggressive groups was found to be similar [12]. Moreover, in other small-scale studies, the findings of the studies were also inconsistent [13,14,15,16,18,19,22].

A recent network meta-analysis reported that ezetimibe did not lead to a significant reduction in CIMT [16]. Consistent with this result, the present study also showed that ezetimibe did not lead to a significant reduction in CIMT (Figure 2). However, the results of subgroup analyses demonstrated the effects of ezetimibe on CIMT. When subgroup analysis according to the presence of FH was performed, CIMT was significantly decreased in the ezetimibe/statin group compared to that in the statin alone group in patients with non-FH, but the CIMT of patients with FH was not affected significantly (Figure 3A). Since patients with FH usually take high doses of statins from an early age, lipid-lowering treatment is often performed before trial initiation. In addition, CIMT is known to increase with age [23,24]. The average age of the participants in the Kastelein study was 45.9, which was younger than that of other RCTs, so the baseline CIMT of the participants in the Kastelein study was the lowest at 0.7 mm. Thus, the newly started intervention may be limited in reducing CIMT progression [13,25]. Importantly, the administration of ezetimibe led to a statistically significant decrease in CIMT in patients with non-FH, in contrast to that in patients with FH (Figure 3A). A previous meta-analysis showed that ezetimibe did not reduce CIMT significantly [16], and it included trials in which statins of different intensities were compared or were compared with niacin. Therefore, the unique effect of ezetimibe on CIMT was not analyzed. To determine the unique effects of ezetimibe in the present study, in this study, only RCTs that confirmed the unique effects of ezetimibe using the same dose of statin in both intervention and control groups were selected. Therefore, these results suggest that ezetimibe can be used to decrease CIMT in patients with non-FH.

According to the 2018 ACC/AHA guideline on the management of cholesterol level, the target level of LDL-C is <70 mg/dL or ≥50% reduction from baseline in patients with a high risk of atherosclerotic CVD for secondary prevention [26]. More strictly, the 2019 European Society of Cardiology/European Atherosclerosis Society (ESC/EAS) guidelines for the management of dyslipidemia recommend LDL-C level <55 mg/dL or ≥50% reduction from baseline in such patients for secondary prevention [1]. In both guidelines, administration of ezetimibe was recommended if the LDL-C target level was not attained even after using the maximum tolerable dose. As was expected in the guidelines, the subgroup analysis in the present study categorized by type of prevention revealed a significant reduction in CIMT with ezetimibe/statin for secondary prevention (Figure 3B). Though there is limited evidence regarding the use of ezetimibe for primary prevention [22], Ouchi et al. showed that ezetimibe reduced the occurrence of primary outcomes (sudden cardiac death, myocardial infarction, or stroke) in patients aged ≥ 75 years with an elevated LDL-C level without a history of coronary disease (HR 0.66 and *p* = 0.002) [17]. However, in the present study, there was no significant difference in CIMT reduction for primary prevention. In two RCTs that used ezetimibe for primary prevention, the study by Kastelein et al. could not conclude that ezetimibe had no effect in primary prevention because ezetimibe had a restricted effect in the form of CIMT reduction in patients with FH and the sample size of the study by Kastelein et al. was more than 12 times larger than that of the study by Kinouchi et al. [13,27]. Therefore, further studies are needed to identify the method of choice for primary prevention with ezetimibe.

In this present study, the LDL-C level was significantly lower with the use of an ezetimibe/statin combination than with the use of statin monotherapy, but TG and HDL-C levels showed no significant difference between ezetimibe/statin and statin alone groups (Figure 4). These results suggest that ezetimibe reduces the LDL level, leading to a decrease in CIMT. Possible mechanisms are as follows. Statins reduce LDL-C levels by inhibiting β-Hydroxy β-methylglutaryl-CoA (HMG-CoA), but increase LDL-C absorption in the intestine through a compensation effect [28]. After that, inhibition of increased intestinal cholesterol absorption through the addition of ezetimibe can lead to a decrease in LDL-C levels, resulting in a decrease in CIMT in the non-FH group. Consistent with the current results, a meta-analysis of 11 RCTs involving 5206 patients showed that the ezetimibe combination resulted in a 14.60-fold LDL-C reduction [29]. However, in subgroup analysis for comparing an ezetimibe/atorvastatin combination with atorvastatin alone, no significant difference in HDL-C level was evident (MD: 0.22 and *p* = 0.358) [13,27]. In addition, although LDL reduction in the FH group was greater than that in the non-FH group (Figure 4), CIMT was not reduced. The reason may be that the initial CIMT baseline level was already low (Table 1).

When all studies were represented as a forest plot (Figure 2), the value of I^2^ was 74%, indicating that the heterogeneity of the effect size between studies was very large. Therefore, sensitivity analysis and meta-regression analysis was conducted to explain the heterogeneity of this effect size. When the results of Kastelein’s study were excluded in the sensitivity analysis, it was confirmed that the heterogeneity of the studies greatly decreased (I^2^ = 10%). Unlike other studies, the Kastelein research targeted patients with FH. It is known that FH patients have high LDL levels from a young age, so the time to reach coronary artery disease is shortened, resulting in early myocardial infarction. In order to confirm that the heterogeneity of this study was indicated by the clinical features of FH, meta-regression analysis was performed with covariates. The regression model was significant in baseline CIMT and LDL (Figure S3), suggesting that baseline CIMT and high LDL, which are the characteristics of FH, may contribute to the heterogeneity of effect sizes between studies. However, further studies of the analysis of meta-regression are required because meta-regression is generally not considered when there are fewer than ten studies.

According to the recently published 2019 ESC/EAS guidelines for the management of dyslipidemias, carotid plaque detection is superior to measurement of CIMT as a CV event predictor [1]. This is because plaque primarily reflects atherosclerosis and is related to the overall atherosclerotic burden in the coronary vascular bed [30]. Meanwhile, CIMT is thought to reflect several morphologic processes and the presence of cardiovascular risk factors [27]. Thus, CIMT can be useful in assessing death, myocardial infarction, and stroke in patients with cardiovascular risk factors [9,31,32]. Moreover, CIMT can be an indicator for early evaluation of systemic atherosclerosis in patients without current plaques because it is found at an earlier stage than plaques [33]. Finally, CIMT may be useful for assessing cardiovascular risk in specific patients groups whose risk cannot be estimated using risk factors in general because it represents the atherosclerotic process of the overall effect of various genetic and environmental factors [34]. Taken together, it is meaningful to measure not only plaque but also CIMT for primary and secondary prevention of myocardial infarction and stroke.

### Strength and Limitation

We included RCTs that used the same statins in both intervention and control groups to determine the unique effects of ezetimibe on CIMT. To the best of our knowledge, this is the first meta-analysis to confirm the unique effect of ezetimibe on CIMT. Second, in this study, subgroup analyses were performed to examine the effects of genetics, and type of prevention on the effects of ezetimibe. We found that ezetimibe can decrease CIMT in patients with non-FH and coronary heart disease.

However, the limitations of the study must also be considered. First, after screening, we only included five trials. Since few studies were included, the reliability of the results cannot be confirmed. In particular, the results of the FH group included only one research, so more studies are needed in the future. Nonetheless, this study suggests the possibility of using ezetimibe to reduce CIMT in non-FH patients. Second, the clinical trials included in the analyses had high heterogeneity although the heterogeneity was reduced when patients with FH were excluded (Figure S2). Third, the overall quality of RCT studies is poor, so the results of the meta-analysis may be biased and incorrect although the inclusion of low quality RCTs does not imply that this meta-analysis is low quality. Finally, in this study, there was a lack of common factors such as history of smoking, alcohol consumption, C reactive protein, and blood pressure that might explain the change in CIMT.

## 5. Conclusions

Lipid-lowering agents such as ezetimibe are recommended in patients with uncontrolled hyperlipidemia and at a high risk of atherosclerotic cardiovascular disease. Especially, CIMT is a surrogate marker of atherosclerosis and a predictor of cardiovascular and cerebral events. The present study showed that the use of LDL-C lowering therapy with ezetimibe led to a significant reduction in CIMT in patients with non-FH and secondary prevention. These findings suggest that the efficacy of ezetimibe may vary among patient groups, with potential CIMT reduction benefits in certain subpopulations. This study will contribute to a better understanding of ezetimibe’s effects and may help inform individualized treatment decisions based on patient characteristics.

## Figures and Tables

**Table 1:** Baseline characteristics of the included studies.

First Author Year	Country	Study Period (Year)	Mean Age (I/C)	Study Population (Prevention Type)	I (mg) (N)	C (mg) (N)	Mean CIMT (I/C) (mm)	LDL-C (I/C) (mg/dL)	TG (I/C) (mg/dL)	HDL-C (I/C) (mg/dL)
Kastelein 2008 [13]	The Netherlands	2	46.1/45.7	FH (primary)	E10 + S80 (322)	S80 (320)	0.70 ± 0.13 /0.69 ± 0.13	319.0 ± 0.7 /317.8 ± 66.1	157.0/160.0	46.7 ± 11.3 /47.4 ± 13.2
Kinouchi 2013 [17]	Japan	1	55.2/53.4	HC (primary)	E10 + F20 (28)	F20 (26)	1.2 ± 0.5 * /1.2 ± 0.8 *	159.0 ± 21.0 /156.0 ± 20.0	144.0/149.0	54.0 ±12.0 /54.0 ± 16.0
Luo 2014 [15]	China	1	66.3/67.2	Elderly patients with HC and CHD (secondary)	E10 + A20 (40)	A20 (44)	1.26 ± 0.24 /1.23 ± 0.25	126.5 ± 13.9 /128.0 ± 17.8	202.0 ± 42.5 /208.2 ± 56.7	45.2 ± 14.7 /45.6 ± 17.8
Luo 2016 [18]	China	1	60.8/61.6	CHD (secondary)	E10 + A20 (74)	A20 (74)	1.27 ± 0.08 /1.26 ± 0.10	138.1 ± 14.7 /136.1 ± 17.8	219.7 ± 39.0 /226.8 ± 56.7	45.2 ± 15.5 /46.0 ± 17.8
Wang 2017 [19]	China	1	58.0/58.0	CHD and T2DM (secondary)	E10 + A20 (51)	A20 (49)	1.26 ± 0.43 /1.27 ± 0.44	136.5 ± 33.6 /133.4 ± 29.0	74.3 ± 7.4 /73.9 ± 8.1	52.1 ± 9.2 /56.0 ± 4.0

* Maximum; I: intervention group, statin with ezetimibe; C: control group, statin alone; N: number; CIMT: carotid intima-media thickness; LDL-C: low-density lipoprotein cholesterol; TG: triglyceride; HDL-C: high-density lipoprotein cholesterol; FH: familial hypercholesterolemia; HC: hypercholesterolemia; CHD: coronary heart disease; T2DM: type 2 diabetes mellitus; E: ezetimibe; S: simvastatin; F: fluvastatin; and A: atorvastatin.

## Data Availability

Data are contained within the article.

## Supplementary Materials

The following supporting information can be downloaded at: https://www.mdpi.com/article/10.3390/medicina59111980/s1, Figure S1: Risk of bias; Figure S2: Sensitivity analysis of affecting on CIMT when omitting Kastelein et al., 2008.; Figure S3: Meta-regression analysis with (A) baseline CIMT, (B) LDL, (C) TG, and (D) HDL as covariates; Table S1: PRISMA Checklist; Table S2: Search strategy for meta-analysis; Table S3: A detailed list of excluded studies and reasons for exclusion; Table S4: Clinical or laboratory adverse events. Table S5: Sensitivity analysis of individual trial effect on CIMT.
