# Supplementary material for: Effect of Combination Therapy with Ezetimibe and Statins versus Statin Monotherapy on Carotid Intima-Media Thickness: A Systematic Review and Meta-Analysis of Randomized Controlled Trials
Source: Medicina (Kaunas). 2023 Nov 10;59(11):1980. doi: 10.3390/medicina59111980 (PMC10673457; doi:10.3390/medicina59111980)
Supplement: Supplementary file 1 [file medicina-59-01980-s001.zip › medicina-2680902-supplementary.pdf]

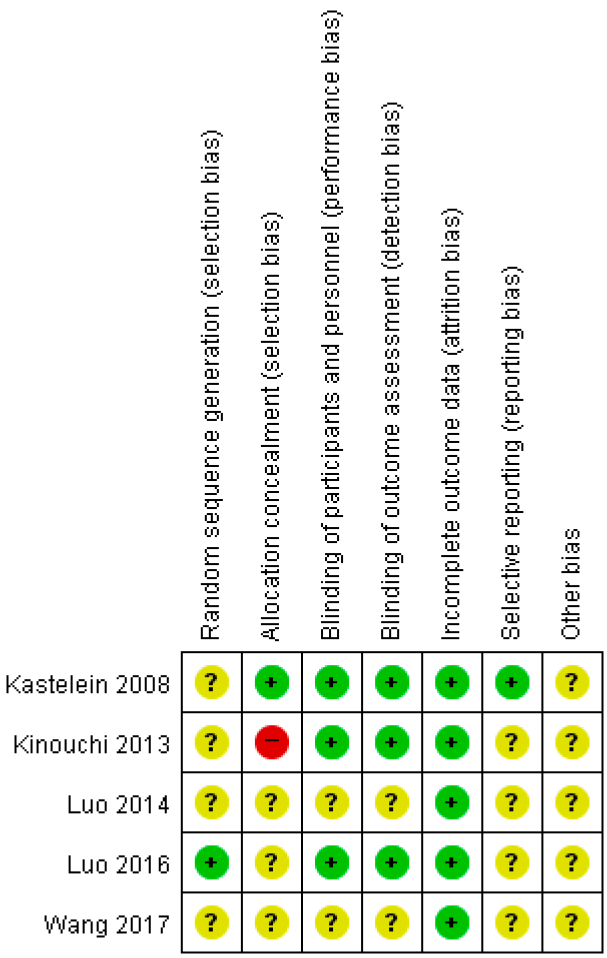

Figure S1. Risk of bias

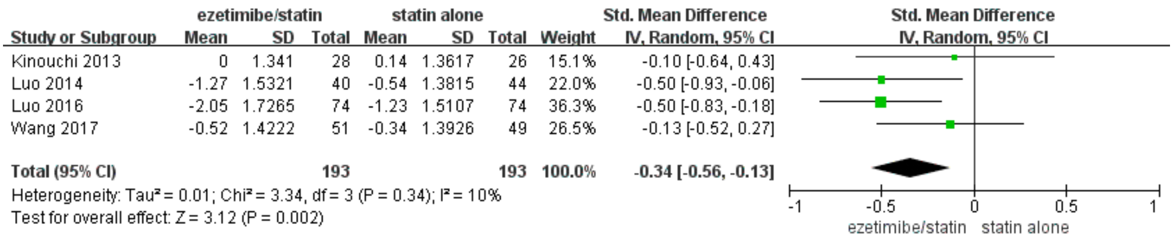

Figure S2. Sensitivity analysis of affecting on CIMT when omitting Kastelein et al, 2008.

CIMT: carotid intima media thickness; SMD: standard mean difference; CI: confidence interval; I^2: I-squared

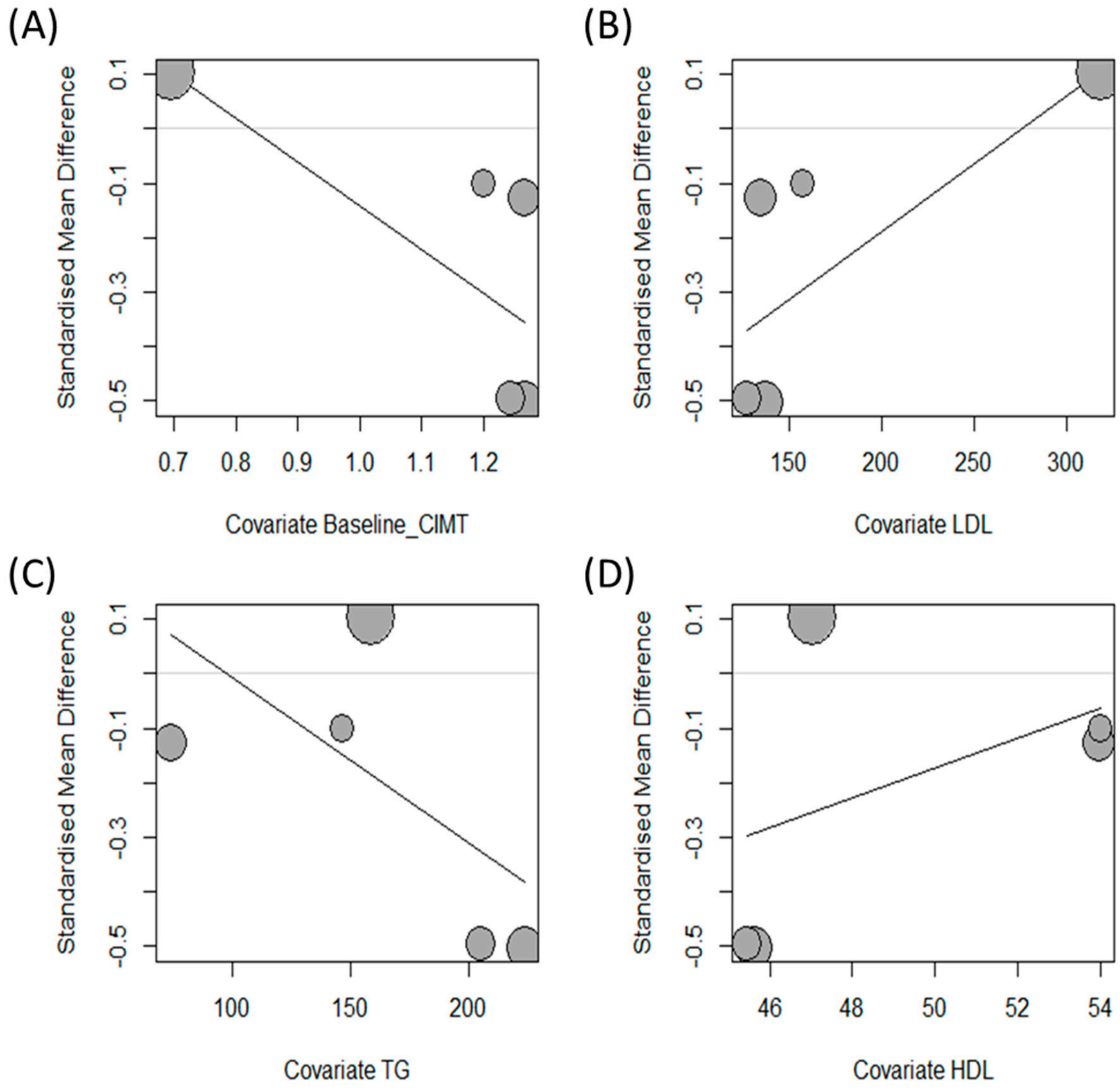

Figure S3. **Meta-regression analysis with (A) baseline CIMT, (B) LDL, (C) TG, and (D) HDL as covariates**

Table S1. PRISMA Checklist

Section/topic	#	Checklist item	Reported on page #
**TITLE**			
Title	1	Identify the report as a systematic review, meta-analysis, or both.	P1
**ABSTRACT**			
Structured summary	2	Provide a structured summary including, as applicable: background; objectives; data sources; study eligibility criteria, participants, and interventions; study appraisal and synthesis methods; results; limitations; conclusions and implications of key findings; systematic review registration number.	P1
**INTRODUCTION**			
Rationale	3	Describe the rationale for the review in the context of what is already known.	P2
Objectives	4	Provide an explicit statement of questions being addressed with reference to participants, interventions, comparisons, outcomes, and study design (PICOS).	P2
**METHODS**			
Protocol and registration	5	Indicate if a review protocol exists, if and where it can be accessed (e.g., Web address), and, if available, provide registration information including registration number.	No registration
Eligibility criteria	6	Specify study characteristics (e.g., PICOS, length of follow-up) and report characteristics (e.g., years considered, language, publication status) used as criteria for eligibility, giving rationale.	P2-3
Information sources	7	Describe all information sources (e.g., databases with dates of coverage, contact with study authors to identify additional studies) in the search and date last searched.	P2, S2 table

Search	8	Present full electronic search strategy for at least one database, including any limits used, such that it could be repeated.	S1 Table
Study selection	9	State the process for selecting studies (i.e., screening, eligibility, included in systematic review, and, if applicable, included in the meta-analysis).	P2
Data collection process	10	Describe method of data extraction from reports (e.g., piloted forms, independently, in duplicate) and any processes for obtaining and confirming data from investigators.	P2-3
Data items	11	List and define all variables for which data were sought (e.g., PICOS, funding sources) and any assumptions and simplifications made.	P3
Risk of bias in individual studies	12	Describe methods used for assessing risk of bias of individual studies (including specification of whether this was done at the study or outcome level), and how this information is to be used in any data synthesis.	P3, S1 Figure
Summary measures	13	State the principal summary measures (e.g., risk ratio, difference in means).	P3
Synthesis of results	14	Describe the methods of handling data and combining results of studies, if done, including measures of consistency (e.g., I^2) for each meta-analysis.	P3
Risk of bias across studies	15	Specify any assessment of risk of bias that may affect the cumulative evidence (e.g., publication bias, selective reporting within studies).	P3, S1 Figure
Additional analyses	16	Describe methods of additional analyses (e.g., sensitivity or subgroup analyses, meta-regression), if done, indicating which were pre-specified.	P3
**RESULTS**			
Study selection	17	Give numbers of studies screened, assessed for eligibility, and included in the review, with reasons for exclusions at each stage, ideally with a flow diagram.	P3-4, S3 Table

Study characteristics	18	For each study, present characteristics for which data were extracted (e.g., study size, PICOS, follow-up period) and provide the citations.	P4, Table 1
Risk of bias within studies	19	Present data on risk of bias of each study and, if available, any outcome level assessment (see item 12).	P3, S1 Figure
Results of individual studies	20	For all outcomes considered (benefits or harms), present, for each study: (a) simple summary data for each intervention group (b) effect estimates and confidence intervals, ideally with a forest plot.	P5-8
Synthesis of results	21	Present results of each meta-analysis done, including confidence intervals and measures of consistency.	P5-8
Risk of bias across studies	22	Present results of any assessment of risk of bias across studies (see Item 15).	S1 Figure, P11
Additional analysis	23	Give results of additional analyses, if done (e.g., sensitivity or subgroup analyses, meta-regression [see Item 16]).	P5-9, Figure 3, S4 Table, S2 Figure.
**DISCUSSION**			
Summary of evidence	24	Summarize the main findings including the strength of evidence for each main outcome; consider their relevance to key groups (e.g., healthcare providers, users, and policy makers).	P9, P11
Limitations	25	Discuss limitations at study and outcome level (e.g., risk of bias), and at review-level (e.g., incomplete retrieval of identified research, reporting bias).	P11
Conclusions	26	Provide a general interpretation of the results in the context of other evidence, and implications for future research.	P11
**FUNDING**			
Funding	27	Describe sources of funding for the systematic review and other support (e.g., supply of data); role of funders for the systematic review.	P11

Table S2. Search strategy for meta-analysis

	**PubMed**	**26 Jan 2023**
I	Ezetimibe [MeSH] OR (1-(4-fluorophenyl)-(3R)-(3-(4-fluorophenyl)-(3S)-hydroxypropyl)-(4S)-(4-hydroxyphenyl)-2-azetidinone) OR Ezetimib OR Ezetimibe OR Ezetrol OR SCH 58235 OR 58235, SCH OR SCH-58235 OR SCH58235 OR Zetia	4,256
O	Carotid Intima-Media Thickness[Mesh] OR Carotid Intima-Media Thickness OR Carotid Intima Media Thickness OR Intima-Media Thickness, Carotid	14,041
	I & O	56
	**Cochrane**	**26 Jan 2023**
I	Ezetimibe [MeSH] OR Ezetimib OR Ezetrol OR SCH 58235 OR 58235, SCH OR SCH-58235 OR SCH58235 OR Zetia OR 1 (4 fluorophenyl) (3R) (3 (4 fluorophenyl) (3S) hydroxypropyl) (4S) (4 hydroxyphenyl) 2 azetidinone	908
O	Carotid Intima-Media Thickness [MeSH] OR Carotid Intima-Media Thickness OR Carotid Intima Media Thickness OR Intima-Media Thickness, Carotid	2,130
	I & O	17
	**Embase**	**26 Jan 2023**

I	'ezetimibe'/exp OR '1 (4 fluorophenyl) 3 [3 (4 fluorophenyl) 3 hydroxypropyl] 4 (4 hydroxyphenyl) 2 azetidinone' OR 'ezetimib' OR 'ezetimibe' OR 'ezetrol' OR 'sch 58235' OR 'sch58235' OR 'zetia'	13,470
O	'carotid intima-media thickness'/exp OR 'carotid intima media thickness' OR 'carotid intima-media thickness' OR 'intima-media thickness, carotid'	13,590
	I & O	136

Table S3. A detailed list of excluded studies and reasons for exclusion

No	Author, Publication year	Study title	Reasons for exclusion
1	Benetos, 2021	Carotid Artery Temperature Reduction with Statin Therapy in Patients with Familial Hyperlipidemia Syndromes	Non RCT
2	Rubba 2017	Causative mutations and premature cardiovascular disease in patients with heterozygous familial hypercholesterolaemia	Non RCT
3	Danielson, 2013	Reduction in carotid intima-media thickness after pancreatic islet transplantation in patients with type 1 diabetes	Non RCT
4	Delialis, 2022	Remnant cholesterol and atherosclerotic disease in high cardiovascular risk patients. Beyond LDL cholesterol and hypolipidemic treatment	Non RCT
5	Fleg, 2008	Effect of statins alone versus statins plus ezetimibe on carotid atherosclerosis in type 2 diabetes: the SANDS (Stop Atherosclerosis in Native Diabetics Study) trial	Non RCT
6	Burggraaf, 2018	Progression of subclinical atherosclerosis in subjects with rheumatoid arthritis and the metabolic syndrome	Non RCT
7	Wang, 2022	Implications of Ezetimibe in Combination with Low- to Moderate-Intensity Atorvastatin Adjuvant Aspirin Therapy for Cerebrovascular Disease	No data of interest
8	Masiá, 2014	Long-term effects of an intensive intervention in HIV-infected patients with moderate-high atherosclerotic cardiovascular risk	No data of interest
9	Amarenco, 2020	Carotid Atherosclerosis Evolution When Targeting a Low-Density Lipoprotein Cholesterol Concentration <70 mg/dL After an Ischemic Stroke of Atherosclerotic Origin	No data of interest
10	Davidson, 2014	Effects of fenofibric acid on carotid Intima-Media thickness in patients with mixed dyslipidemia on atorvastatin therapy: Randomized, placebo-controlled study (FIRST)	No data of interest
11	Masiá, 2009	A pilot randomized trial comparing an intensive versus a standard intervention in stable HIV-infected patients with moderate - High cardiovascular risk	No data of interest
12	Rehberger Likozar, 2022	Smoking and diabetes attenuate beneficial effects of PSCK9 inhibitors on arterial wall properties in patients with very high lipoprotein (a) levels	No data of interest
13	Taylor 2012	Paradoxical progression of atherosclerosis related to low-density lipoprotein reduction and exposure to ezetimibe	No data of interest
14	Toth 2012	Changes in prescription patterns before and after reporting of the Ezetimibe and Simvastatin in Hypercholesterolemia Enhances Atherosclerosis Regression trial (ENHANCE) results and expected effects on low-density lipoprotein-cholesterol reduction	No data of interest
15	Russell 2010	Achieving lipid targets in adults with type 2 diabetes: the Stop Atherosclerosis in Native Diabetics Study	No data of interest

16	Villines 2010	The ARBITER 6-HALTS Trial (Arterial Biology for the Investigation of the Treatment Effects of Reducing Cholesterol 6-HDL and LDL Treatment Strategies in Atherosclerosis): final results and the impact of medication adherence, dose, and treatment duration	No data of interest
17	Meaney 2009	The VYtorin on Carotid intima-media thickness and overall arterial rigidity (VYCTOR) study	No data of interest
18	Taylor 2009	Extended-release niacin or ezetimibe and carotid intima-media thickness	No data of interest
19	Devine 2007	Design and rationale of the ARBITER 6 trial (Arterial Biology for the Investigation of the Treatment Effects of Reducing Cholesterol)-6-HDL and LDL Treatment Strategies in Atherosclerosis (HALTS)	No data of interest
20	Kastelein 2005	Comparison of ezetimibe plus simvastatin versus simvastatin monotherapy on atherosclerosis progression in familial hypercholesterolemia. Design and rationale of the Ezetimibe and Simvastatin in Hypercholesterolemia Enhances Atherosclerosis Regression (ENHANCE) trial	No data of interest
21	Vera-Lastra 2015	Effect of pravastatine plus ezetimibe on carotid intima media thickness in patients with lupus erythematosus	No control group
22	Morita 2014	Renal and vascular protective effects of ezetimibe in chronic kidney disease	No control group

Table S4. Clinical or laboratory adverse events

Adverse events	Kastelein 2008	Kinouchi 2013	Luo 2014	Luo 2016	Wang 2017
Discontinuation	↔<br>(C: 34/363, E: 29/357)	↔<br>(C: 0/26, E: 0/28)	↔<br>(C: 0/44, E: 0/40)	↔<br>(C: 0/74, E: 0/74)	↔<br>(C: 0/49, E: 0/51)
Cardiovascular events	↔<br>(C: 7/363, E:10/357)	N.D.	↔<br>(C: 0/44, E: 0/40)	↔<br>(C: 9/74, E: 9/74)	N.D.
Liver<br>(AST/ALT)	↔	↔	↔	↔	↔
Muscle<br>(CK, myopathy, or muscle weakness)	↔	↔	↔	↔	↔
Kidney<br>(Creatinine)	N.D.	↓	N.D.	N.D.	N.D.
Heart<br>(EKG)	↔	↔	N.D.	N.D.	N.D.
Inflammation<br>(CRP)	↓	N.D.	↔	N.D.	↔

↔: No significant difference between control and experiment groups; ↓: Significant decrease between control and experiment groups; N.D.: not determined or unknown; Cardiovacular events: nonfatal myocardial infarction, nonfatal stroke, coronary revascularization, cardiovascular death, or stroke during a follow-up; C: control group (statin alone); E: experiment group (statin+ezetimibe); AST: aspartate aminotransferase; ALT: alanine aminotransferase; CK: creatine kinase; CRP: C reactive protein; EKG: electrocardiogram

Table S5. Sensitivity analysis of individual trial effect on CIMT

Omitting Study	SMD	Lower limit	Upper limit	Z value	P value
- [Kastelein et al, 2008]	-0.34	-0.56	-0.13	3.12	0.002
- [Kinouchi et al, 2013]	-0.23	-0.58	0.12	1.30	0.19
- [Luo et al, 2014]	-0.14	-0.46	0.17	0.90	0.37
- [Luo et al, 2016]	-0.11	-0.39	0.16	0.79	0.43
- [Wang et al, 2017]	-0.23	-0.61	0.14	1.23	0.22

CIMT: carotid intima media thickness; SMD: standard mean difference.
